# Social isolation and diurnal cortisol patterns in an ageing cohort^☆^

**DOI:** 10.1016/j.psyneuen.2013.07.002

**Published:** 2013-11

**Authors:** Mai Stafford, Mike Gardner, Meena Kumari, Diana Kuh, Yoav Ben-Shlomo

**Affiliations:** a MRC Unit for Lifelong Health and Ageing, London, UK; b School of Social and Community Medicine, University of Bristol, Bristol, UK; c Department of Epidemiology and Public Health, UCL, London, UK

**Keywords:** NSHD, Psychosocial, HPA axis, Bereavement, Social isolation

## Abstract

**Background:**

Social isolation may operate as a psychosocial stressor which disrupts functioning of the hypothalamic–pituitary–adrenocortical axis.

**Methods:**

Using data from the MRC National Survey of Health and Development, we tested whether living alone, not being married and social network size were associated with diurnal cortisol patterns at 60–64 years. We hypothesised that recent onset compared with long-term isolation would be more strongly associated with cortisol awakening response, cortisol decline over the day and evening cortisol. Models were adjusted for sex, smoking, body mass index, alcohol intake, psychological distress and financial difficulties.

**Results:**

Those widowed within the last three years had a 36% (95%CI 6%, 73%) higher night time cortisol than those who were currently married. Those newly living alone also had a higher night time cortisol and flatter diurnal slope than those living with others.

**Conclusion:**

Independently of multiple behavioural and psychosocial correlates, recent onset of social isolation is related to diurnal cortisol patterns that increase the risk of morbidity and mortality.

## 1 Introduction

Social isolation is associated with coronary heart disease mortality and morbidity (Lett et al., 2005; Ramsay et al., 2008; Holt-Lunstad et al., 2010; Udell et al., 2012). It has been proposed that social isolation may operate as a psychosocial stressor which affects coronary heart disease risk through disrupted functioning of the hypothalamic–pituitary–adrenocortical (HPA) axis (Cacioppo et al., 2002; Hackett et al., 2012). Activation of the HPA axis causes an increase in cortisol, which can induce physiological changes in response to the stressor. In healthy individuals cortisol is high on waking, peaks at around 30–45 min post-waking and then falls gradually to reach its lowest point at around midnight (Kumari et al., 2010a). However, repeated or chronic activation of the HPA axis is associated with negative health outcomes including coronary heart disease (Davey Smith et al., 2005; Kumari et al., 2011). Disrupted HPA-axis functioning may be indicated by a flatter than average slope between morning and night time, and a high night time cortisol level (Adam and Kumari, 2009). Both high and low morning cortisol and high or blunted cortisol awakening response (that is, the difference between the waking and peak cortisol) have been associated with poorer health (Adam and Kumari, 2009; Gardner et al., 2011, 2013). In large-scale epidemiological studies, diurnal cortisol has typically been measured in saliva samples as cortisol in saliva accurately reflects levels of free, biologically active cortisol in blood, often with the aim of examining its relationship to social environmental exposures, such as social isolation.

Social isolation may be measured objectively by its structural form (captured by various indicators including living alone, having a small network size and not being married) and as perceived isolation (typically captured by reported feelings of loneliness). Objective isolation is a risk factor for perceived isolation though correlations are moderate in size in older adults and the two forms of isolation appear to relate independently to physical health (Cornwell and Waite, 2009). Individuals may not feel isolated despite lacking structural social connections. Earlier studies provide some support for an association between objective social isolation and cortisol patterns. Concurrently, socially isolated study members of an occupational cohort study, that is having infrequent or no contact with friends and relatives or living alone, were found to have a higher cortisol awakening response (CAR), independently of perceived social isolation (Grant et al., 2009). Based on measurements collected over four days, female students who took part in a greater number of activities with others had a steeper decline in cortisol throughout the day (Stetler et al., 2004). A flatter slope was seen for women who were divorced or widowed compared with those currently married in a small study of cancer patients (Sephton et al., 2000). Others have failed to find an association between objective indicators of social isolation and cortisol response (Turner-Cobb et al., 2000; Lai et al., 2012). To date, although social isolation is more prevalent among older compared with younger people, its association with cortisol patterns has not been established in large, general population samples. In addition, studies have predominantly focused on concurrent social isolation whereas the timing of the onset of isolation may moderate its association with cortisol output. Meta-analysis demonstrated that morning cortisol was lower and daily cortisol output higher for chronically stressed compared with control groups but that the magnitude of these differences reduced with increasing time since stressor onset (Miller et al., 2007). Included studies were based on people who had experienced war, abuse, bereavement, job loss, caregiving and disaster though the impact of recent onset versus long term social isolation was not examined.

Cortisol patterns differ by sex (Kumari et al., 2010a) and a number of behavioural and psychosocial factors that may covary with social isolation including smoking (Kumari et al., 2010a), obesity (Kumari et al., 2010b), alcohol intake (Badrick et al., 2008), psychological distress (Miller et al., 2007) and socioeconomic disadvantage (Kumari et al., 2010c; Agbedia et al., 2011), which are important to take into account in any observational study. The current study addresses two questions: (i) is there a cross-sectional association between multiple indicators of social isolation and diurnal cortisol, and (ii) does time since onset of isolation moderate the association between isolation and cortisol? It extends existing literature by examining these associations in a large, general population sample of older people and by considering the role of recent versus longer-term social isolation. On the basis of previous studies of social isolation and of other indicators of psychosocial stress, we hypothesised that greater isolation would be associated with a blunted cortisol pattern. Since objective isolation may, to some extent, be a matter of individual choice and because individuals adapt psychologically to life events that may increase the risk of isolation including bereavement and divorce (Luhmann et al., 2012), we further hypothesised that recent onset isolation would be more strongly associated with a blunted cortisol pattern than long-term isolation.

## 2 Methods

The oldest British birth cohort, the MRC National Survey of Health and Development (NSHD), completed its 23rd follow-up between 2006 and 2011 when study members were aged 60–64 years (Kuh et al., 2011). The NSHD is based on a social class stratified sample of 5362 births of all singleton births that occurred within marriage in a week in March 1946 in England, Scotland and Wales. Study members are white Caucasian; recruitment to the study pre-dated major immigration into Britain. The previous main data collection was at 53 years. The 60–64 year data collection consisted of a postal questionnaire followed by clinical assessment by research nurses in one of six clinical research facilities across the UK or at home, during which consent for salivary cortisol sample collection was obtained. A total of 2229 out of 2856 (78%) eligible study members (those known to be alive and with a known address in England, Scotland or Wales) took part in a clinic (*n* = 1690) or home visit (*n* = 539). Those opting for a home visit were more likely to be obese, a lifelong smoker, with lower educational attainment, and occupying a lower socioeconomic position compared with those attending the clinic (Stafford et al., 2013). Invitations were not sent to those who had died (*n* = 778), who were living abroad (*n* = 570), had previously withdrawn from the study (*n* = 594) or had been lost to follow-up (*n* = 564). Ethical approval for the study was obtained from the Greater Manchester Local Research Ethics Committee and the Scotland A Research Ethics Committee. Written, informed consent was obtained from the study member for each component of data collection.

Study members were asked to provide three saliva samples in salivettes at 9–9.30 pm on the evening of the clinic or home visit and at usual waking time and waking +30 min the following day. They were trained in the protocol for cortisol collection by the research nurse and requested not to drink for 30 min before taking the sample and not to smoke, eat or brush their teeth until after the evening sample and after the waking +30 min sample. Deviations from the protocol and stressful events before the sample was taken were self-reported, though actual waking time (if different from time of taking the first cortisol sample) was not captured. Salivettes and reports were returned by post. Samples were frozen and subsequently assayed by radioimmunoassay in a laboratory (Dresden) specialising in high through-put cortisol assays (Kirschbaum and Hellhammer, 1989). The cortisol collection was not initially included in the protocol and so 348 study members living in the Manchester area of the UK, who constituted the feasibility sample at this sweep, were not invited to take part in this element of the study.

### 2.1 Social isolation

Information on current marital status, year of separation or widowhood, household composition and number of friends and relatives seen at least monthly was obtained from postal questionnaires in 2006–11 and at the previous follow-up interview in 1999. Amongst those whose marital status was divorced in 2006–11, the median time since divorce was 8 years (25th centile 6 years, 75th centile 12 years). Corresponding figures for time since widowhood were 4 years (2 years, 6 years). Time since divorce or widowhood was dichotomised at three years for pragmatic reasons to include sufficient numbers in the newly widowed group. Remarried study members were included in the currently married group. Those who were living alone in 2006–11 but not in 1999 (the “newly living alone”) were distinguished from those living alone at both occasions (assumed to be long-term living alone). Frequency of informal social contact was classified as seeing zero to two (low social contact), or more than three friends or relatives at least monthly. Those with low social contact in 2006–11 but not in 1999 were distinguished from those with long-term low social contact.

### 2.2 Health behaviour and psychosocial covariates

Current smokers were distinguished from ex and never smokers. Measured height and weight were used to derive body mass index (BMI), included here as categories since non-linear associations between BMI and cortisol have been found (Kumari et al., 2010b). The 28-item General health questionnaire (GHQ-28) was used as a continuous score to capture symptoms of psychological distress. Self-reported financial difficulties were used to identify those who had problems managing, were managing adequately and were managing well.

### 2.3 Statistical analysis

Study members on steroid medication (*n* = 21) and cortisol sample values >100 nmol/L (*n* = 11) were removed from the analysis. Waking and waking +30 min cortisol samples collected before 5.00 am and after noon (*n* = 55) and night time samples collected after 2.15 am (*n* = 3) were removed from the analysis as these may indicate shift workers.

Three aspects of the diurnal cortisol pattern were examined. Night time cortisol was log-transformed due to its skewed distribution. The diurnal slope was calculated by subtracting the cortisol at night time from that at waking and is expressed as change per hour since waking. The cortisol awakening response (CAR) was calculated by subtracting the cortisol at waking from estimated cortisol at waking +30 min. The latter was calculated as fitted cortisol at +30 min plus the person-level residual from a linear regression model and was used to correct for differences in lapsed time between 1st and 2nd samples but those who collected the second sample at 60 or more minutes after the waking sample were not included.

There was moderate overlap in the three indicators of social isolation and so they were included as independent variables in separate linear regression models. All models were adjusted for sex and for eating, smoking, drinking or being stressed immediately prior to the sample collection. Adjustment for regular smoking, body mass index, alcohol intake, psychological distress and financial difficulties did not materially alter the estimates and so we present only estimates which are adjusted for these behavioural and psychosocial covariates. Models for CAR and diurnal slope were additionally adjusted for waking time. There was no evidence of sex by social isolation interactions and so results for men and women are presented together.

To avoid loss of power due to missing covariates and reduce the impact of bias due to differential non-response, we undertook a full imputation maximum likelihood analysis (FIML). This approach borrows information from the observed portion of the data under the assumption of data being missing at random (that is, the missing observations are random conditional on the set of covariates included in the model).

## 3 Results

At 60–64 years study members who were not included in the analytical sample because of incomplete data had a flatter slope, were less likely to be married and more likely to be living alone or have a small social network size compared with included study members. Evening cortisol level and CAR did not differ significantly for those who were and were not included.

Median waking time was 7.13 am (25th centile 6.30 am, 75th centile 7.50 am and 90% of study members woke between 5.55 am and 9 am). The median time for collecting the evening sample was 9.20 pm (9.05 pm, 9.30 pm, 90% of study members sampling between 9.00 pm and 10.45 pm). Compared with women, men had a higher mean waking cortisol, steeper diurnal slope and smaller CAR (*t*-test *p* < 0.001) but no differences in waking + 30 or evening cortisol (*t*-test *p* > 0.4; Table 1). There were no differences in mean waking, waking + 30, or evening cortisol for those who had a home versus clinic visit, but those assessed at clinic had a steeper diurnal slope (−1.22 versus −1.11, *p* = 0.008) and a smaller CAR (6.09 versus 7.41, *p* = 0.04). We therefore adjusted the following analyses of diurnal slope and CAR for whether the study member was assessed at clinic or home. Approximately 7% of the sample was newly living alone and almost 10% had experienced a reduction in their social network since the previous data collection at 53 years.

Current smoking and financial difficulties were more prevalent amongst those who were living alone, not currently married or had a small social network compared with those living with others, married or with medium to large social networks (Table 2).

Current smokers had higher night time cortisol than non-smokers (data available on request). Those with a BMI of between 30 and 34 kg/m^2^ had higher night time cortisol whereas the decline over the day was less steep for those with BMI of 35 kg/m^2^ or more compared with those who had BMI between 25 and 29 kg/m^2^. Night time cortisol was higher amongst those who had problems managing financially compared with those who were managing well.

After adjustment for sex, smoking, body mass index, psychological distress, financial difficulties and non-adherence to cortisol collection protocol, those newly living alone at 60–64 years had mean log night time cortisol 0.19 (95%CI 0.05, 0.34) nmol/L higher than those not living alone (Table 3, first column of estimates). This can be interpreted as those newly living alone having a night time cortisol level 21% (95%CI 5%, 40%) higher than those living with others on the original, untransformed scale (calculated as (exp(0.19) − 1) × 100%). Those who had been widowed within the last three years had a mean log night time cortisol level 0.31 (95% CI 0.06, 0.55) nmol/L higher than those who were currently married. This translates to a 36% (95% CI 6%, 73%) higher night time cortisol on the original scale. Additional analysis revealed that those who had been widowed within the last three years had a 23% higher night time cortisol level than those who had been widowed for more than three years though the confidence interval was wide (95% CI 12% lower to 74% higher).

**Figure 1:**
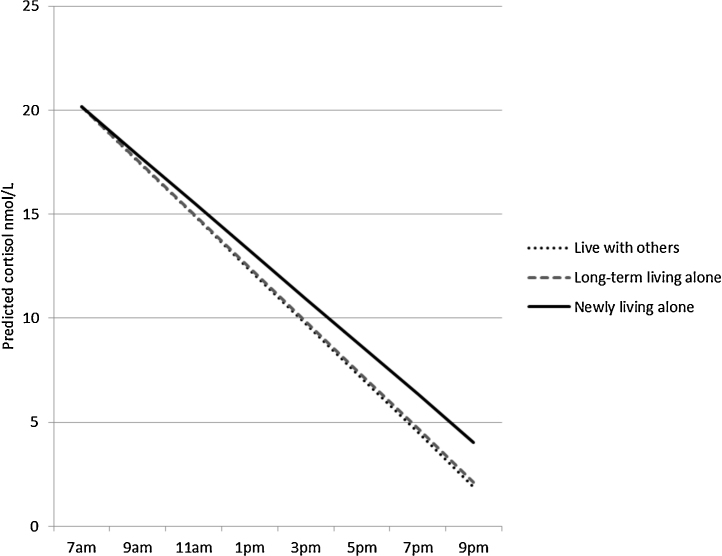
Cortisol slope by change in living arrangements. Lines indicate decline in cortisol over the day for those newly living alone, long-term living alone, and currently living with others.

**Figure 2:**
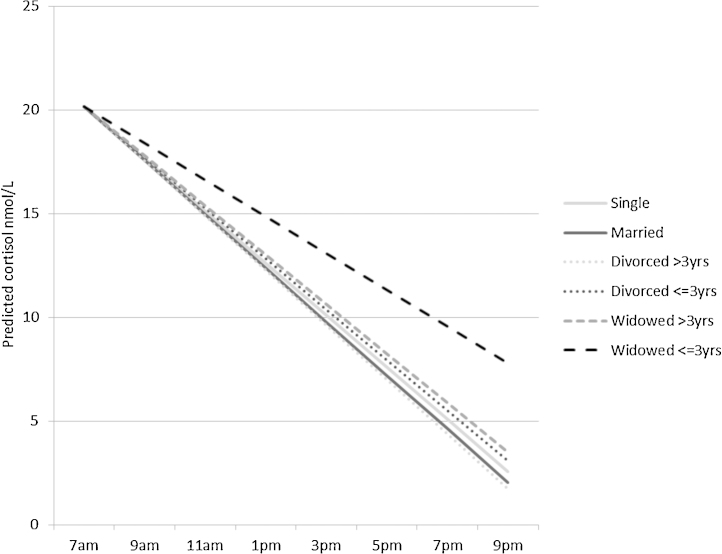
Cortisol slope by change in marital status. Lines indicate decline in cortisol over the day for those who are single, married, divorced, and widowed.

Those newly living alone had a flatter slope than those living with their partner, and being widowed in the last three years was also associated with a flatter slope compared with being married (Table 3, second column of estimates, Figs. 1 and 2). As can be seen from Figs. 1 and 2, the slope in the reference group is negative in sign. The regression estimate of 0.40 (95%CI 0.11, 0.68) represents the difference in slope for those widowed in the last three years compared with married study members. It indicates that the drop in cortisol between waking and evening is less negative, that is less steep, than in the reference group. In additional analyses, differences in diurnal slope between those who had been widowed within the last three years compared to more than three years did not attain statistical significance (0.27 (95% CI – 0.11, 0.64) nmol/L/h). Those who had been separated/divorced for more than three years had a lower CAR and those who had been widowed for more than three years had a higher CAR compared with the currently married group.

Among those newly living alone, 27.9% had been widowed within the last ten years and 6.8% within the last three years (Table 4). Although this indicated only moderate overlap between newly living alone and newly widowed, further analysis revealed that when both social isolation exposures were included simultaneously the associations between newly living alone and both night time cortisol and cortisol slope were attenuated and became statistically non-significant. On the other hand, the associations between being newly widowed and night time cortisol and cortisol slope remained (data available from the authors).

## 4 Discussion

We have shown that cortisol patterns in early older age differ by living arrangements and marital status. In particular, higher night time cortisol and flatter diurnal slope, both of which are associated with increased mortality and disease risk, were seen for study members who were living alone and those who were widowed. These findings partially agree with those from a recent study in an occupational cohort which found a greater total cortisol output but also higher CAR in the group with the highest levels of social isolation (Grant et al., 2009). They also complement a meta-analysis indicating that exposure to a range of chronic psychosocial stressors including bereavement, unemployment, combat, abuse, and caregiving is associated with a higher cortisol level in the evening, a flatter slope through the day and higher total output (Miller et al., 2007).

We also set out to examine whether any link between social isolation and cortisol differed for recent onset versus long-term isolation. Study members who had been recently widowed had somewhat higher night time cortisol levels and flatter slopes than long-term widows, although the differences in the estimates did not achieve formal statistical significance. Similarly, higher night time cortisol and flatter slopes were seen among those who were newly living alone compared to those who had been living alone at the current and previous sweep. We acknowledge that these are arbitrary classifications of time since isolation onset and that we were limited by relatively low statistical power for investigating moderation but our findings provide some support for the hypothesis that the association between a chronic stressor and cortisol depends on the time since onset of stress. Longitudinal studies indicate a recovery in mental wellbeing in the months following widowhood or divorce (Luhmann et al., 2012). Psychological and behavioural adaptation to such life events may be accompanied by changes in diurnal cortisol profiles towards patterns associated with lower morbidity and mortality risk. As far as we are aware, repeat measures of social isolation have not been examined in relation to cortisol patterns, though the MIDUS study based on a nationally representative US sample was used to examine social strain on two occasions over a 10-year follow-up (Friedman et al., 2012). The authors found that the group perceiving high strain at both occasions had the flattest diurnal cortisol and found no differences in diurnal cortisol between those perceiving high strain at the second wave only compared with no high strain. That study thus captured cumulative exposure to the psychosocial stress of negative social interactions whereas we suggest that, in the current study, onset of social isolation may be a better marker of psychosocial stress than is chronic isolation.

*A priori*, we had expected to see cortisol patterns indicating disrupted HPA-axis functioning for study members who had been divorced. An earlier study showed that women currently going through or anticipating divorce had higher evening cortisol than control women not undergoing marital stress (Powell et al., 2002). We did not find evidence to support this though our estimates for study members who experienced divorce within the last three years were not reliable due to very small sample size in this group. Possibly divorce is part of a long-term process of relationship breakdown and the event of divorce may not in this case be a good indicator of onset of psychosocial stress. Our study found that the direction of the association with the CAR also differed for separated/divorced and widowed study members. We may speculate that the event of divorce may not capture emotional loss to the same extent as widowhood, although the latter may signal the end of caring for an ill spouse.

We did not find social network size to be associated with cortisol patterns, in common with some earlier work (Turner-Cobb et al., 2000; Lai et al., 2012). One possibility for this null finding is that our measure of social network size does not capture the particular features of social relationships that are of most relevance for HPA-axis functioning. Previous work suggests that social relationships that involve regular or daily contact may be particularly important for cortisol rhythms (Stetler and Miller, 2008).

Objective social isolation may be linked to cortisol patterns through multiple pathways including reduced opportunity to confide and more limited access to financial and practical resources, both of which may increase the risk of psychological distress, poor health behaviours and cortisol (Prince et al., 1997; Chida and Steptoe, 2009; Laaksonen et al., 2009). Social isolation may also result in an absence of health-promoting social influence (Berkman et al., 2000). We found associations between cortisol patterns and smoking, obesity, and financial difficulties, each of which were found to be more prevalent among those who are isolated compared with the socially integrated. However, these covariates did not materially attenuate the identified associations between social isolation and cortisol suggesting that the behavioural and psychological factors that we have captured here are not important mediators. Stetler et al. (2004) propose that regular activities undertaken in the presence of other people help to programme diurnal rhythms, including diurnal cortisol patterns. In support of their hypothesis, they demonstrate that regular social activities including eating and exercising are associated with steeper diurnal decline in healthy adults. We did not include these behaviours in the current analyses. Neither did we measure physical touch, which may buffer stress response although experimental studies are inconsistent on this point (Ditzen et al., 2007; Holt-Lunstad et al., 2008). Sleep may also be implicated in the association between isolation and cortisol (Cacioppo et al., 2002; Steptoe et al., 2004; Kumari et al., 2009). The association of sleep duration and disturbance with cortisol patterns is potentially bi-directional (Gillin et al., 1972; Leproult et al., 1997), though whether newly sleeping alone would be associated with a more or less disrupted HPA axis may depend on several factors including the prior quality of the marital relationship and other features of the partner's sleep (Troxel et al., 2007). It was beyond the scope of this study to examine these associations however.

Some other limitations must be acknowledged. We lacked detailed information on the timing of living alone and on changes to living arrangements between data sweeps, and had low statistical power for exploring time since isolation as a moderator. We did not find interactions between social isolation indicators and sex though numbers isolated when broken down by sex are small and so we cannot rule out the possibility of important interactions. Previous studies have been similarly underpowered and/or examined women only (Sephton et al., 2000; Stetler et al., 2004; Grant et al., 2009). Remarried study members constituted 16.3% of the married group but were not distinguished from the continually married here. To the extent that previous marital dissolution has a long-term influence on cortisol patterns, these results may somewhat underestimate the differences between widowed and continually married study members. Diurnal cortisol was captured over a single 24 h period though it varies from day to day. It is possible that the stresses associated with completing a clinical assessment on the same day as collection of the evening cortisol sampling affected cortisol values. In addition to the potential mediating role of sleep discussed earlier, we note that diurnal rhythms are anchored to a person's usual sleeping and waking schedules (Adam and Kumari, 2009). We did not capture usual waking and sleeping times and so cannot rule out the possibility that differences between usual sleep schedules and the sleep schedule during cortisol collection may vary according to levels of social isolation and may thus confound our results. Cortisol was not assessed in the previous data collection so we have not been able to directly test whether the association between isolation and cortisol varies by time since isolation onset within an individual. The use of FIML models is based on the assumption, which cannot be verified, that missing data patterns are missing at random. Whilst we have demonstrated a relationship between objective isolation and cortisol patterns, we do not currently have data that enable us to determine whether this is independent of, or mediated by, subjective loneliness (Adam et al., 2006; Grant et al., 2009; Doane and Adam, 2010; Hackett et al., 2012). As with all longitudinal studies, there has been some attrition. Compared with those who responded at 53 years but not or not fully at 60–64 years, responders at 60–64 years tended to have higher educational attainment and adult occupational social class, and were less likely to be long-term smokers, obese or physically inactive (Stafford et al., 2013). Thus we may not have been able to include those with the poorest cortisol profiles in the current analyses.

Our longitudinal findings in this representative general population cohort of older people add to the accumulating body of evidence linking social isolation to HPA axis function which is implicated in the biology of ageing (Sapolsky et al., 1986). Independently of multiple behavioural and psychosocial correlates, recent onset of social isolation is related to diurnal cortisol patterns that increase the risk of morbidity and mortality.

## Role of funding source

MS and DK are supported by the UK Medical Research Council. MG was supported from a New Dynamics of Ageing grant (RES-353-25-0001). MK is partially funded by the ESRC (RES-596-28-0001). The funders had no role in study design, data collection and analysis, decision to publish, or preparation of the manuscript.

## Conflicts of interest

None to declare.

## Figures and Tables

**Table 1:** Cortisol values by demographic, behavioural and psychological characteristics.

	All (max *n* = 1791)	Men (max *n* = 840)	Women (max *n* = 951)
Mean (sd)			
Waking (nmol/L)	20.2 (10.2)	21.9 (11.2)	18.8 (9.0)
Waking + 30 min (nmol/L)	26.2 (11.7)	26.5 (12.2)	26.0 (11.2)
Evening; geometric mean (nmol/L)	2.59 (2.65)	2.58 (2.64)	2.60 (2.65)
Diurnal slope (nmol/L/h)	−1.19 (0.74)	−1.30 (0.81)	−1.09 (0.66)
Cortisol awakening response (nmol/L)	6.49 (11.91)	4.98 (12.28)	7.62 (11.44)

% (*n*) study members			
Living arrangements			
Living with others	87.8 (1426)	90.5 (687)	85.3 (739)
Long-term living alone	5.4 (88)	4.6 (35)	6.1 (53)
Newly living alone	6.8 (11)	4.9 (37)	8.6 (74)
Marital status			
Single never married	3.7 (60)	4.2 (32)	3.2 (28)
Currently married	79.5 (1291)	82.7 (627)	76.6 (664)
Separated/divorced >3 years	10.3 (168)	9.4 (71)	11.2 (97)
Separated/divorced ≤3 years	0.6 (10)	0.9 (7)	0.4 (3)
Widowed >3 years	3.9 (63)	1.6 (12)	5.9 (51)
Widowed ≤3 years	2.0 (33)	1.2 (9)	2.8 (24)
Social network			
Large network	84.3 (1438)	79.7 (628)	88.3 (810)
Long-term small network	5.9 (101)	7.2 (57)	4.8 (44)
Reduction in social network	9.7 (166)	13.1 (103)	6.9 (63)
Smoking status at 60–64			
Current smoker	10.4 (171)	10.3 (79)	10.5 (92)
Ex-never smoker (reference group)	89.6 (1470)	89.7 (686)	89.5 (784)
Body mass index at 60–64 (kg/m^2^)			
<25	29.4 (525)	24.8 (208)	33.4 (317)
25–29	40.8 (730)	46.8 (392)	35.6 (338)
30–34	21.6 (386)	22.3 (187)	21.0 (199)
35+	8.2 (147)	6.1 (51)	10.1 (96)
Alcohol intake in last week			
None	15.9 (246)	11.4 (86)	20.2 (160)
Moderate^a^	70.9 (1094)	69.2 (520)	72.5 (574)
High^b^	13.2 (204)	19.4 (146)	7.3 (58)
Psychological distress symptoms at 60–64			
0–4 symptoms	86.4 (1504)	90.5 (741)	82.8 (763)
5+ symptoms	13.6 (237)	9.5 (78)	17.3 (159)
Financial difficulties at 60–64			
Problems managing	6.3 (104)	7.0 (54)	5.7 (50)
Managing OK	37.1 (614)	38.7 (300)	35.8 (314)
Managing well	56.6 (935)	54.3 (421)	58.5 (514)

a Men <22 units; women <15 units.

b Men 22+ units; women 15+ units.

**Table 2:** Behavioural and psychosocial factors by social isolation at 60–64 years.

	Live with others	Live alone	Married	Not married	Medium to large network	Small network
Column %						
Current smoker	9.2	18.1‡	8.5	18.2‡	9.5	13.7†
Body mass index 35+ kg/m^2^	7.8	12.0†	7.6	11.4†	8.3	7.5
No alcohol in last week	15.2	17.0	14.4	18.4	16.1	14.4
High alcohol intake in last week	13.5	13.2	13.1	14.8	13.5	11.1
5+ psychological distress symptoms	13.4	11.1	12.6	14.4	13.0	18.0†
Problems managing financially	4.6	12.4‡	3.9	12.9‡	6.0	8.0†
Managing OK	36.1	41.6	35.4	41.5	35.5	45.1

† *p* < 0.05.

‡ *p* < 0.001.

**Table 3:** Multiply adjusted association between social isolation and cortisol patterns. Each social isolation indicator is considered separately (not mutually adjusted).

	Log night time cortisol (log nmol/L)^a^	Diurnal slope (nmol/L/h)^b^	CAR (nmol/L)^c^
Living arrangements at 60–64 (reference “live with others”)			
Long-term living alone	0.12 (−0.04, 0.27)	0.02 (−0.16, 0.19)	−1.05 (−3.94, 1.84)
Newly living alone	**0.19 (0.05, 0.34)**	**0.15 (0.00, 0.31)**	1.70 (−0.81, 4.22)

Marital status at 60–64 (reference “currently married”)			
Single never married	**0.23 (0.05, 0.41)**	0.03 (−0.18, 0.24)	−1.17 (−4.57, 2.24)
Separated/divorced >3 years	0.05 (−0.07, 0.17)	−0.03 (−0.16, 0.17)	**−2.79 (−4.93, −0.65)**
Separated/divorced ≤3 years	−0.19 (−0.64, 0.25)	0.05 (−0.48, 0.57)	−3.62 (−11.80, 4.57)
Widowed >3 years	0.16 (−0.02, 0.34)	0.11 (−0.10, 0.31)	**3.88 (0.56, 7.19)**
Widowed ≤3 years	**0.31 (0.06, 0.55)**	**0.40 (0.11, 0.68)**	0.41 (−4.09, 4.91)

Social network (reference “large social network”)			
Long-term small network	−0.05 (−0.17, 0.07)	0.09 (−0.03, 0.22)	0.76 (−1.36, 2.87)
Reduction in social network	0.00 (−0.14, 0.14)	0.12 (−0.04, 0.27)	0.90 (−1.79, 3.59)

Bold indicates difference from reference significant at 5% level.

a Adjusted for sex, smoking, body mass index, psychological distress score, financial difficulties, non-adherence to protocol.

b Adjusted for sex, waking time, lapsed time since waking, smoking, body mass index, psychological distress score, financial difficulties, clinic or home assessment, non-adherence to protocol.

c Adjusted for sex, waking time, smoking, body mass index, psychological distress score, financial difficulties, clinic or home assessment, non-adherence to protocol.

**Table 4:** Marital status by living arrangements.

	Living with others	Long-term living alone	Newly living alone
*n* with marital status	1398	87	109
Column %			
Single never married	1.4	32.2	8.3
Currently married	92.0	0.0	0.9
Separated/divorced >10 years	4.2	48.3	20.2
Separated/divorced 4–10 years	0.9	0.0	15.6
Separated/divorced ≤3 years	0.3	0.0	3.7
Widowed >10 years	0.5	12.6	10.1
Widowed 4–10 years	0.4	4.6	21.1
Widowed ≤3 years	0.4	2.3	6.8
